# P-1959. Duration and Quantification of Histoplasma capsulatum Antigenuria During Treatment of Histoplasmosis in Patients by Immune Status

**DOI:** 10.1093/ofid/ofaf695.2126

**Published:** 2026-01-11

**Authors:** Samuel M Fallon, Julio C Zuniga-Moya, Patrick B Mazi, Adriana Rauseo, Andrej Spec

**Affiliations:** Washington University School of Medicine in St. Louis, St. Louis, MO; Washington University School of Medicine in St. Louis, St. Louis, MO; Washington University, St Louis, Missouri; Washington University in St. Louis, Saint Louis, MO; Washington University School of Medicine in St. Louis, St. Louis, MO

## Abstract

**Background:**

Despite its classic association with HIV, modern cohorts of histoplasmosis are overwhelmingly in those with non-HIV-associated immunocompromise, such as biologic immunosuppression or solid organ transplantation. However, treatment guidelines are largely extrapolated from 30-year-old studies of patients with HIV.

**Figure:**
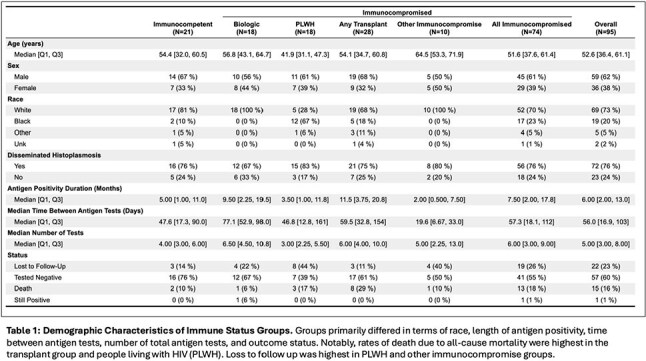

**Figure:**
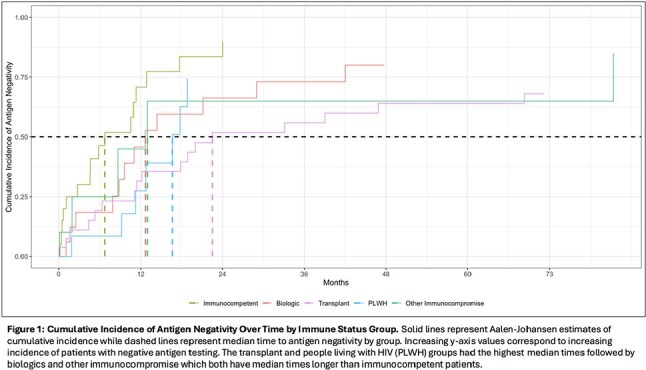

**Methods:**

We conducted a single-center, retrospective study of adult patients with varying immune status diagnosed with histoplasmosis by positive antigen testing and undergoing treatment between 2002 to 2021. Time to *H. capsulatum* antigen negativity was calculated and compared between immune status groups using competing risk analysis. Per-patient, mean antigen levels were compared at 6mo intervals from diagnosis.

**Figure:**
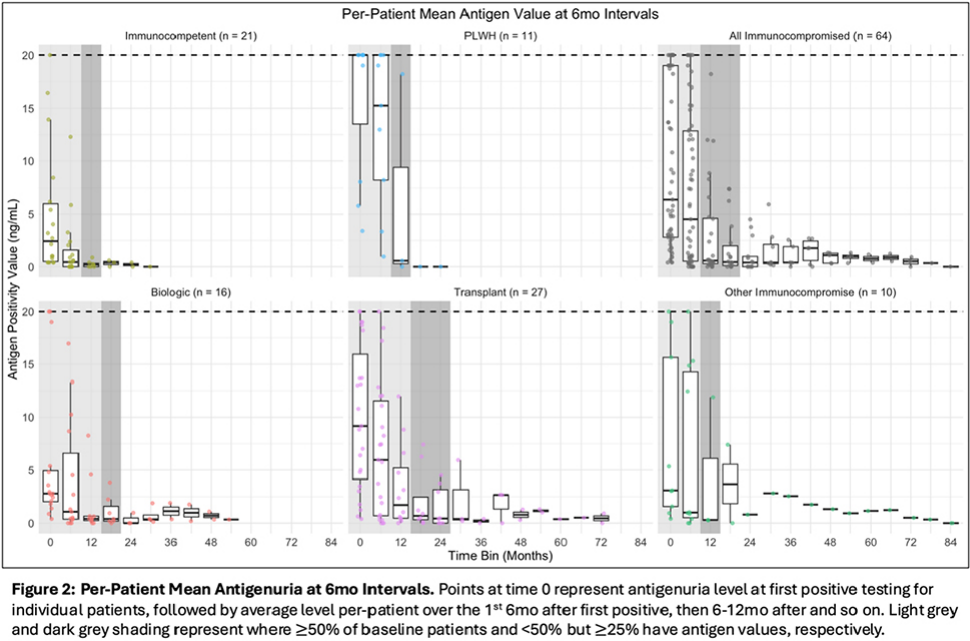

**Figure:**
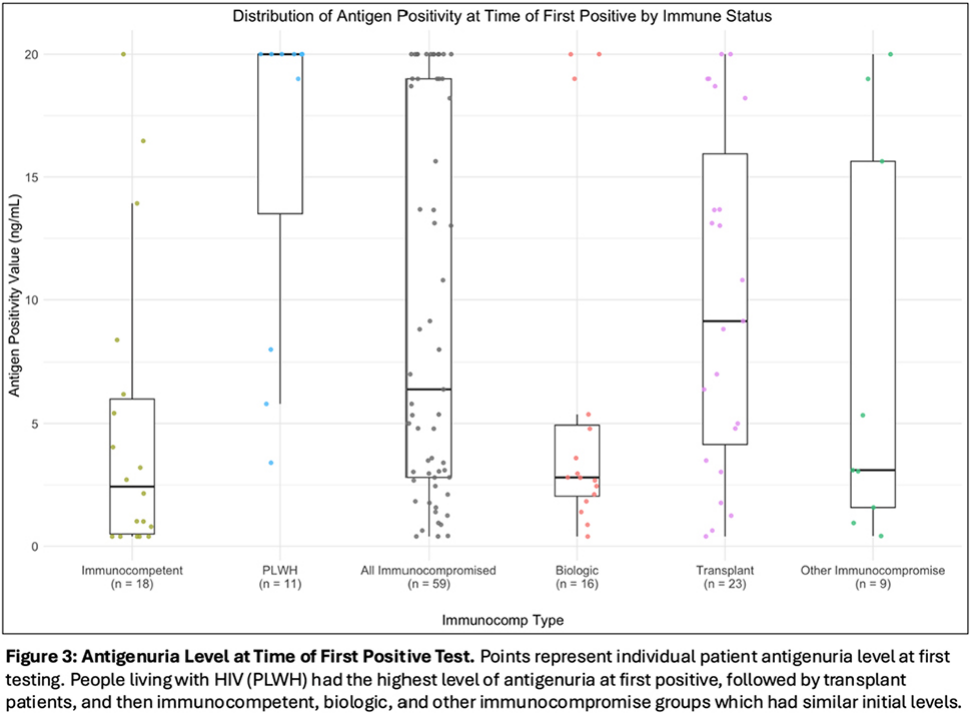

**Results:**

95 patients treated for histoplasmosis had varying immunocompromise: immunocompetent (n=21), biologic immunosuppression (n=18), people living with HIV (PLWH) (n=18), any transplant (n=28), and other (n=10) – primarily prednisone with methotrexate or other disease modifying therapy (7/10). Median time to antigen negativity, accounting for competing risk of death by group was: transplant - 22.7mo, PLWH - 16.9mo, biologics - 12.9mo, other immunocompromise - 13.2mo, and immunocompetent - 6.8mo (Figure 1). Median antigen level at diagnosis was highest in PLWH (20ng/mL [Q1: 13.5, Q3: 20]) and transplant patients (9.2ng/mL [4.1, 15.9]) versus the other groups – immunocompetent: (2.4ng/mL [0.5, 6.0]), biologics: (2.8ng/mL [2.0, 4.9]), other: (3.1ng/mL [1.6, 15.6]) (Figures 2 & 3). Of patients not deceased or lost to follow-up at 24mo, 31.6% (6/19) of transplant patients and 28.6% (4/14) of biologic patients had positive antigen testing versus 0% (0/7) of PLWH, 6.2% (1/16) of immunocompetent patients and 20% (1/5) of other immunocompromise patients.

**Conclusion:**

Median time to antigen negativity was highest in transplant patients and PLWH and intermediate in biologic and other immunocompromise groups relative to immunocompetent patients. More than 80% of patients with antigenuria at 2yrs were in the transplant or biologic groups. Duration and magnitude of antigen positivity varied across immune status, impacting length of treatment for histoplasmosis.

**Disclosures:**

Andrej Spec, M.D., MSCI, Astellas Global Development Pharma, Inc: Grant/Research Support|Cidara: Grant/Research Support|Mayne Pharma: Grant/Research Support|Scynexis: Grant/Research Support

